# Ontogenetic plasticity in cranial morphology is associated with a change in the food processing behavior in Alpine newts

**DOI:** 10.1186/s12983-020-00373-x

**Published:** 2020-11-16

**Authors:** Daniel Schwarz, Nicolai Konow, Laura B. Porro, Egon Heiss

**Affiliations:** 1 Institute of Zoology and Evolutionary Research, Friedrich Schiller University Jena, Erbertstraße 1, D-07743 Jena, Germany; 2 Department of Biological Sciences, University of Massachusetts Lowell, 198 Riverside St, Lowell, MA 01854 USA; 3 Centre for Integrative Anatomy, Department of Cell and Developmental Biology, University College London, London, WC1E 6BT UK

**Keywords:** Salamander, Kinematics, Micro-CT, Functional morphology, Feeding apparatus, Ontogeny, Chewing, Intraoral food processing, Feeding, Flexibility

## Abstract

**Background:**

The feeding apparatus of salamanders consists mainly of the cranium, mandible, teeth, hyobranchial apparatus and the muscles of the cranial region. The morphology of the feeding apparatus in turn determines the boundary conditions for possible food processing (i.e., intraoral mechanical reduction) mechanisms. However, the morphology of the feeding apparatus changes substantially during metamorphosis, prompting the hypothesis that larvae might use a different food processing mechanism than post-metamorphic adults. Salamandrid newts with facultative metamorphosis are suitable for testing this hypothesis as adults with divergent feeding apparatus morphologies often coexist in the same population, share similar body sizes, and feed on overlapping prey spectra.

**Methods:**

We use high-speed videography to quantify the in vivo movements of key anatomical elements during food processing in paedomorphic and metamorphic Alpine newts (*Ichthyosaura alpestris*). Additionally, we use micro-computed tomography (μCT) to analyze morphological differences in the feeding apparatus of paedomorphic and metamorphic Alpine newts and sort them into late-larval, mid-metamorphic and post-metamorphic morphotypes.

**Results:**

Late-larval, mid-metamorphic and post-metamorphic individuals exhibited clear morphological differences in their feeding apparatus. Regardless of the paedomorphic state being externally evident, paedomorphic specimens can conceal different morphotypes (i.e., late-larval and mid-metamorphic morphotypes). Though feeding on the same prey under the same (aquatic) condition, food processing kinematics differed between late-larval, mid-metamorphic and post-metamorphic morphotypes.

**Conclusions:**

The food processing mechanism in the Alpine newt changes along with morphology of the feeding apparatus during ontogeny, from a mandible-based to a tongue-based processing mechanism as the changing morphology of the mandible prevents chewing and the tongue allows enhanced protraction. These results could indicate that early tetrapods, in analogy to salamanders, may have developed new feeding mechanisms in their aquatic environment and that these functional innovations may have later paved the way for terrestrial feeding mechanisms.

## Background

Most salamanders switch from a feeding larval- to a post-metamorphic stage during ontogeny via metamorphosis [1–3]. A recent study suggests that in species undergoing metamorphosis, parts of the skull associated with feeding develop faster and more independently from the rest [4]. This in turn suggests that the form and function of skeletal elements associated with feeding may be more flexible. From an ontogenetic perspective, this flexibility is particularly useful for metamorphic species that change their food spectrum over the course of their lives due to the transition from aquatic to terrestrial environments. However, some salamanders do not undergo metamorphosis but instead attain sexual maturity while retaining larval traits [5, 6]. This somatic developmental arrest is referred to as paedomorphosis, and is particularly common among salamanders [7–10]. In some salamander species, individuals can either undergo or skip metamorphosis (i.e., facultative paedomorphosis) [8, 11], resulting in paedomorphic and metamorphosed adults co-populating similar niches of a habitat whilst differing in morphology (i.e., heterochronic morphotypes). Prior studies have hypothesized that, due to their different morphologies, heterochronic morphotypes differ in their feeding performance (capture success-rate) and feeding behavior [12–14]. Behavioral studies have shown that paedomorphs tend to have greater aquatic prey capture performance [12, 14], but surprisingly, despite diverging prey capture performance and major differences in head morphology, there are only minor differences in prey capture kinematics between heterochronic morphotypes [13–16].

Prey capture is followed by intraoral behaviors, which can include distinct processing and transport cycles. Similar to prey capture kinematics, transport kinematics do not seem to differ significantly between larval and post-metamorphic morphotypes [17–20]. However, it is unclear whether intraoral processing kinematics follow the same pattern as capture and transport. First, although recent evidence suggests that intraoral food processing is more common in salamanders than previously thought [21–23], processing remains little studied in salamanders compared to other taxa [24, 25]. Second, processing might be affected more from differing feeding apparatus morphologies than capture and transport. This latter point becomes more evident if we consider changes in the structure, position and number of the teeth [26–29]; structural changes of the hyobranchial apparatus (i.e., developing from a gill-bearing to a tongue-bearing apparatus) [14, 15, 30–32]; changes in the muscular and ligamentous suspension of the hyobranchial apparatus [33–35]; morphological changes of mandible and skull [15, 36–38]; as well as dramatic muscular reorganization [39, 40] during metamorphosis in salamanders. All of the aforementioned characteristics impact intraoral food processing kinematics in salamanders [21, 23].

Food processing in salamanders involves a mix of structural and functional traits seen in fishes and amniotes [21]. Salamanders, being lissamphibians, are especially interesting from an evolutionary point of view because of their phylogenetic position near the base of the tetrapod radiation, with lissamphibians being considered the extant sister-group of amniotes. As a result, salamanders are critical to our understanding of the functional evolution of tetrapods, because they might retain many basal features in the musculoskeletal system [41, 42], including a broad and flat skull [43, 44], and a similarly robust anatomy of the hyobranchial apparatus [45]. The lissamphibian metamorphosis enables the experimental investigation of developmental water-land transitions in recent tetrapods [41] – as an analogy to the evolutionary water-land transitions of early tetrapods.

Accordingly, our objectives in the present study are: a) to compare the intraoral food processing kinematics and feeding apparatus morphologies of the heterochronic morphotypes of the Alpine newt (*Ichthyosaura alpestris*) and b) to propose a possible evolutionary scenario of the prey-processing behavior in early tetrapods. We quantify how changes in form of the feeding apparatus can induce shifts in feeding kinematics. We hypothesize that while prey capture and transport kinematics are similar between paedomorphic and post-metamorphic Alpine newts, intraoral processing kinematics will differ between heterochronic morphotypes.

## Results

### Functional morphology of the feeding apparatus

Detailed descriptions of the cranial anatomy of *Ichthyosaura alpestris* and other salamandrids can be found elsewhere [26, 32, 46–52] and we focus on structures relevant for processing and on specific differences between morphotypes.

#### Cranial osteology

**Fig. 1:**
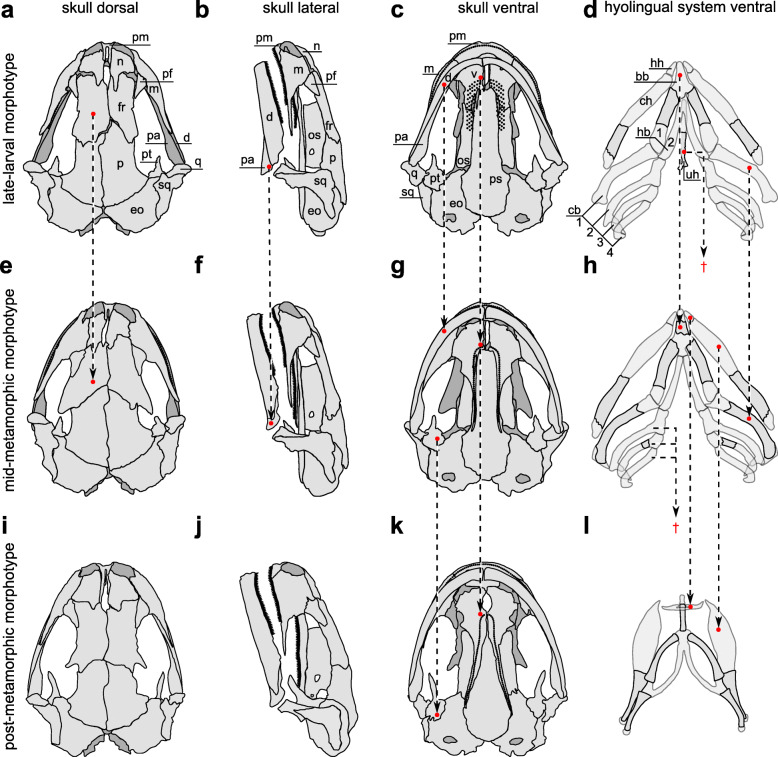
Skeletal morphology of the feeding apparatus of different morphotypes in *I. alpestris*. **a-d** (row 1) late-larval morphotype (LLM), **e**-**h** (row 2) mid-metamorphic morphotype (MMM), **i**-**l** (row 3) post-metamorphic morphotype (PMM). Abbreviations: (bb) basibranchial, (cb 1–4) ceratobranchial 1–4, (ch) ceratohyal, (d) dentary, (eo) exoccipital, (fr) frontal, (hh) hypohyal (also referred to as radial), (hb 1–2) hypobranchial 1–2, (m) maxilla, (n) nasal, (os) orbitosphenoid, (p) parietal, (pa) prearticular, (pf) prefrontal, (pm) premaxilla, (ps) parasphenoid, (pt) pterygoid, (q) quadrate, (sq) squamosal, (uh) urohyal, (v) vomer. Arrows connecting different morphotypes (rows) highlight significant structural differences. Arrows ending in the space between morphotypes marked with † indicate the reduction of the structure

**Fig. 2:**
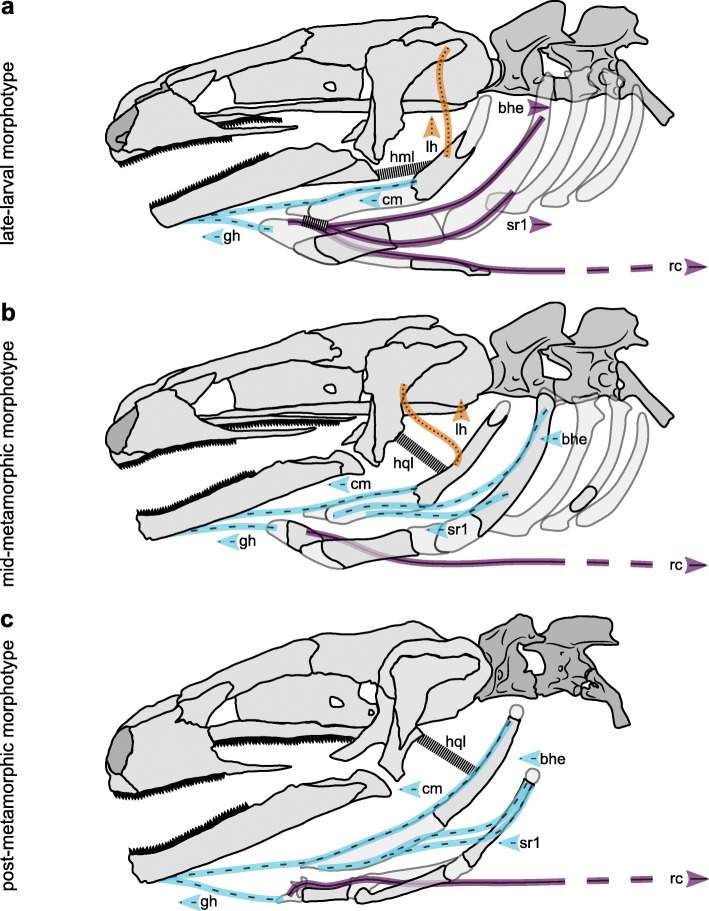
Muscular morphology of the feeding apparatus of three different morphotypes of *I. alpestris*: (**a**) the late-larval morphotype (LLM), (**b**) the mid-metamorphic morphotype (MMM), (**c**) the post-metamorphic morphotype (PMM). Muscles: (bhe) branchiohyoideus externus, (cm) ceratomandibularis, (gh) geniohyoid, (lh) levator hyoideus, (rc) rectus cervicis and (sr1) subarcualis rectus 1. Ligaments: (hml) hyomandibular ligament and (hql) hyoquadrate ligament. The directional effect of each muscle on the movement of the tip of the hyobranchial apparatus (i.e., the basibranchial) is encoded by the arrows, protractors (blue and dashed), retractors (purple and solid), and elevators (orange and dotted). Note that there is no direct hyobranchial elevator in the PMM. Depression of the hyobranchial system is achieved by a combination of rectus cervicis activity and the ligamentous and muscular suspension of the hyobranchial skeleton to the skull. Please note that the course of the ligaments was obtained from other morphological descriptions [33, 35, 48] and could not be verified in this study

The feeding apparatus of the Alpine newt consists of an osseous skull and mandible, and a complex, partially cartilaginous hyobranchial system (i.e., hyobranchial in larval or hyolingual in metamorphosed salamanders, respectively) (see Fig. 1) and prominent muscles (Fig. 2). We group the paedomorphic (p) and metamorphic (m) specimens into three distinct morphotypes: (i) late-larval (p), (ii) mid-metamorphic (p), and (iii) post-metamorphic (m), based on their developmental state. The anterior skull plates of the late-larval morphotype (LLM) are largely unfused while in the mid-metamorphic morphotype (MMM) and the post-metamorphic morphotype (PMM) the enlarged frontal bones fill those gaps. The pterygoids of LLM and MMM are relatively small compared to those of the PMM. All morphotypes carry two functional upper jaw systems: the first consists of the tooth bearing maxilla and premaxilla (i.e., "primary" upper jaw), and the second of the tooth bearing vomerine and palatine bones of the mouth roof (i.e., "secondary" upper jaw or palatal jaw).

The palatal dentition pattern of the LLM is U-shaped and the teeth organized in rows, the mandible is slightly V-shaped in ventral view and the functional occlusal surface for the lower jaw dentition is the palate between "primary" and "secondary" upper jaws. The mandibles of MMM and PMM are U-shaped in ventral view and the occlusal surface for the lower jaw are the maxillary teeth of the "primary" upper jaw. The palatal dentition of the MMM and the PMM are distinct as the MMM has a U-shaped single row of denticles and the PMM exhibits a V-shaped single row of denticles.

#### Hyobranchial musculoskeletal anatomy

The hyobranchial apparatus shows the most striking differences between morphotypes. In the LLM, the hyobranchial apparatus is a complex and mainly cartilaginous system with small ossification centers in ceratohyal, hypobranchial and urohyal. The hyobranchial apparatus of MMM shows enlargement of these ossified centers, additional ossification centers in basibranchial, ceratobranchial 1 and 3, as well as the reduction of the urohyal. The hyolingual apparatus of the PMM exhibits a typical morphology for metamorphosed salamandrids. Thus, in the PMM the ceratobranchial 2–4 are reduced and the hypohyals merge to form a buckle around basibranchial (often referred to as the radial).

Our functional descriptions of the hyobranchial apparatus focus on muscles responsible for the main movements of the anterior tip of the hyobranchial system (i.e., the basibranchial). The 3D muscle morphology is considered but the main function of each muscle is assessed from a lateral perspective (i.e., simplified to a 2D movement). More complex inter-hyobranchial movements are likely to occur due to the 3D orientation of the hyobranchial apparatus and its muscles (see for example [53]). The hyobranchial system of all morphotypes forms the attachment site for several major muscles (i.e., six in the LLM and MMM, and five in the PMM). The muscles can be differentiated according to their initial attachment to hyoid arch or branchial arch during ontogeny [54]. The hyoid arch (paired ceratohyals) is connected with the ceratomandibularis (CM) and branchiohyoideus externus (BHE) mucles in all morphotypes, and also with the levator hyoideus (LH) in the LLM and MMM. The CM extends between the ossified area of the ceratohyal and the dentary in all morphotypes, acting as a protractor of the hyobranchial system. The fleshy BHE extends from the lateral side of the postero-dorsal ceratobranchial I to a tendoninous sheet connecting the anterior regions of the hyoid- and branchial arch in the LLM. Because of the ligamentous connection of the ceratobranchial to the mandible (HML), the BHE serves as a retractor of the anterior part of the branchial system while it adduces the anterior tip of the hyobranchial apparatus and the posterior part of ceratobranchial I. In contrast, in the MMM and the PMM, the insertion of the BHE shifted completely to the antero-ventral part of ceratohyal and therefore acts as a protractor of the branchial arch. In the LLM, the LH originates on the dorsal squamosal process and attaches to the upper osseous part of the ceratohyal, while in the MMM the LH originates from the mid-squamosal and attaches to the upper osseous part of ceratohyal. Accordingly, the LH serves as a hyobranchial elevator in LLM and MMM. The LH is missing in the PMM because the LH detaches from the hyobranchial system during development in order to attach to the lower jaw and thus form the depressor mandibulae posterior. Apart from the development of the depressor mandibulae posterior, the cranial muscles for opening and closing the jaw showed no significant differences between the morphotypes.

The branchial arch is connected with geniohyoid (GH), subarcualis rectus 1 (SR1), and rectus cervicis (RC) mucles. The thin GH muscle extends from the basibranchial to the dentary in all morphotypes, thus enabling protraction of the hyobranchial system. A peculiarity in metamorphs is that some fibers of the GH extend from the pericardium to the dentary [47]. In the LLM, the SR1 extends from the antero-ventral side of the cartilaginous ceratobranchial I anteriorly to a tendoninous sheet connecting the anterior regions of the hyoid- and branchial arch. Thus, the SR1 acts similarly as BHE in the LLM, retracting the tip of the hyobranchial system, while in the MMM and the PMM the SR1 extends from the medial part of the ceratobranchial I to the medio-lateral part of the ceratohyal to act as a protractor of the branchial arch. The most prominent muscle of the hyobranchial system in all morphotypes is the RC that originates from the ventral abdominal trunk muscles and inserts onto the basibranchial. Due to its course and the ligament and muscle suspension of the hyobranchial apparatus on the skull (hyomandibular or hyoquadrate ligament and levator hyoideus), the RC facilitates retraction and depression of the hyobranchial apparatus.

### Intraoral food processing

**Fig. 3:**
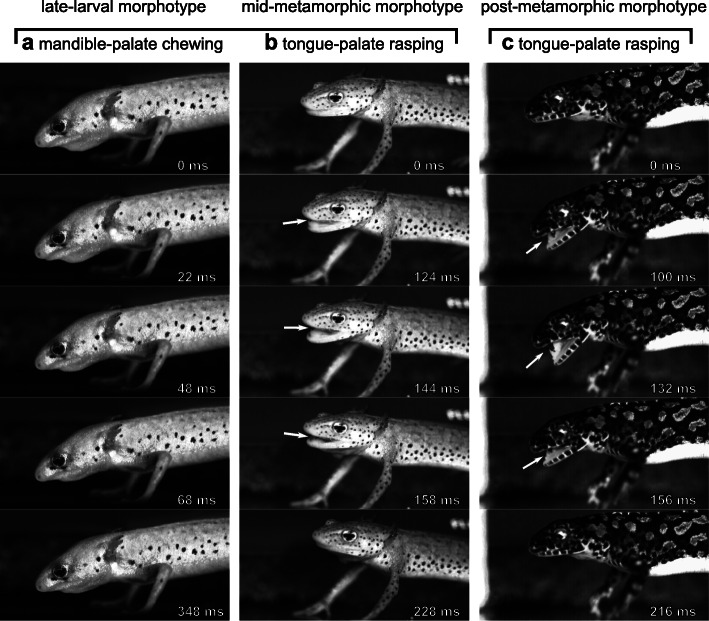
Exemplary snapshots of food processing in three different morphotypes of *I. alpestris*: **a** mandible-palate chewing in late-larval morphotype (LLM), **b** tongue-palate rasping in mid-metamorphic morphotype (MMM), and **c** tongue-palate rasping in the post-metamorphic morphotype (PMM). Note the similar prey processing patterns of mid-metamorphic morphotype and the post-metamorphic morphotype versus the distinct pattern of late-larval morphotype. The arrows point to the position of the prey item when it is visible from the outside

After initial ingestion via suction feeding, one or two transport movements were used by all morphs to position prey prior to a consecutive set of processing cycles. The mean total processing cycles were 5.7 ± 3.2 (mean ± S.D.) for the late-larval, 5.6 ± 2.4 for the mid-metamorphic, and 5.9 ± 2.5 for the post-metamorphic morphotypes. A processing cycle was defined from start of gape opening until the next start of gape opening. Processing involved the cyclical opening and closing of the jaw (i.e., arcuate mandible movement), elevation and depression of the hyobranchial apparatus (i.e., the tongue) and, in the post-metamorphic morphotype only, additional rhythmic flexion and extension of the neck (vertical cranial movement) (Fig. 3c). During these movements, prey debris and haemolymph were occasionally expelled from the oral cavity, indicating that the behavior caused significant prey disintegration. After a processing bout (i.e., a series of processing cycles), water flows induced by hyobranchial movement transported the food backwards, after which it was either repeatedly processed or swallowed.

#### Kinematics of intraoral food processing

**Fig. 4:**
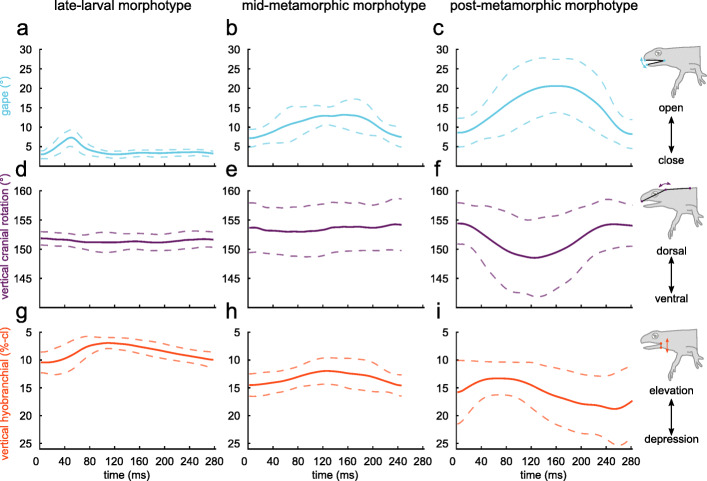
Profiles of the kinematic variables during food processing in three different morphotypes of *I. alpestris*. Kinematic means (dark and bold profiles) ± SD (slender, dashed and pale curves) of synchronous motions plotted in normalized coordinate systems with group-normalized timescales (x-axes) for comparison. Gape (light blue) (**a**, **b**, **c**) and cranial flexion (purple) (**d**, **e**, **f**) in degree, vertical hyobranchial movement (orange) (**g**, **h**, **i**) normalized to cranial length (i.e., %-cl)

Intraoral food processing cycles were clearly distinguishable from food transport in that hyobranchial elevation accompanied gape opening during processing, whereas during transport hyobranchial depression accompanied gape opening. During processing, at the onset of gape opening, the LLM initiated hyobranchial elevation, which continued past peak gape opening and reached its peak coincident with complete gape closure. Then, in a returning motion, the hyobranchial apparatus was depressed while the mouth remained shut (i.e., stationary phase). The MMM started elevating the hyobranchial apparatus at the onset of gape opening. Both movements peaked approximately at the same time, after which simultaneous gape closing and hyobranchial depression (i.e., resetting movements) occurred (Fig. 4b and h). Neither the LLM nor the MMM had stereotypic cranial movements, as indicated by their relative featureless cranial kinematic profiles (Fig. 4d, e). In the PMM gape and vertical cranial flexion peaks were approximately coincident; thus, gape opening and cranial ventroflexion (or head depression) as well as gape closing and cranial dorsoflexion were aligned (Fig. 4c and f). The vertical hyobranchial movement had a ~ 10% phase shift (i.e., delay) from the gape cycle as hyobranchial elevation started at ~ 90% of the preceding gape cycle (compare Fig. 4c and i).

Table 1 shows the kinematic parameters of food processing in the three morphotypes. The stationary gape phase in the LLM clearly differed from the other two morphotypes (compare Fig. 4a with b and c) as did the cranial flexion of the PMM (compare Fig. 4 f with d and e).

**Table 1:** Kinematic parameters of intraoral food processing of three morphotypes of *I. alpestris*

Kinematic variable	Kinematic parameter	Late-larval morphotype (LLM)		Mid-metamorphic morphotype (MMM)		Post-metamorphic morphotype (PMM)	
		Mean ± S.D.	C_V_	Mean ± S.D.	C_V_	Mean ± S.D.	C_V_
Gape	1 Opening (°)	5.35 ± 1.54	0.29	9.82 ± 4.28	0.44	19.30 ± 5.90	0.24
	2 Closure (°)	5.51 ± 1.21	0.22	9.48 ± 4.68	0.49	19.66 ± 5.86	0.23
	3 Opening duration (s)	0.04 ± 0.02	0.45	0.13 ± 0.06	0.48	0.16 ± 0.06	0.32
	4 Closure duration (s)	0.06 ± 0.02	0.36	0.11 ± 0.04	0.38	0.12 ± 0.06	0.44
	5 Closure acceleration (10^−3^ deg/s^2^)	21.40 ± 6.15	0.29	12.25 ± 6.99	0.57	18.75 ± 10.47	0.45
	6 Open-close duration (s)	0.10 ± 0.03	0.25	0.24 ± 0.07	0.27	0.28 ± 0.07	0.21
	7 stationary duration (s)	0.17 ± 0.08	0.45	n/a	n/a	n/a	n/a
	8 Cycle duration (s)	0.28 ± 0.09	0.33	0.24 ± 0.07	0.27	0.28 ± 0.07	0.21
Vertical cranial flexion	9 Ventral (°)	n/a	n/a	n/a	n/a	12.61 ± 6.51	0.39
	10 Dorsal (°)	n/a	n/a	n/a	n/a	12.30 ± 6.46	0.40
	11 Ventral duration (s)	n/a	n/a	n/a	n/a	0.10 ± 0.05	0.41
	12 Dorsal duration (s)	n/a	n/a	n/a	n/a	0.17 ± 0.07	0.33
	13 Cycle duration (s)	n/a	n/a	n/a	n/a	0.28 ± 0.07	0.21
Vertical hyobranchial movement	14 Elevation (%-cl)	4.29 ± 1.99	0.46	4.93 ± 1.47	0.30	10.57 ± 6.18	0.53
	15 Depression (%-cl)	3.87 ± 1.34	0.35	5.43 ± 1.50	0.28	12.46 ± 5.64	0.36
	16 Elevation duration (s)	0.08 ± 0.02	0.24	0.14 ± 0.06	0.45	0.13 ± 0.05	0.34
	17 Depression duration (s)	0.20 ± 0.09	0.44	0.10 ± 0.03	0.35	0.14 ± 0.05	0.31
	18 Cycle duration (s)	0.28 ± 0.10	0.35	0.23 ± 0.07	0.28	0.27 ± 0.07	0.23

Abbreviations: *S.D.* Standard deviation, *C*_*V*_ Coefficient of variation and n/a not applicable. Note that parameters 6 and 8 are identical for both MMM and the PMM. This is because both MMM and PMM lack a stationary phase during processing (parameter 6), so that opening and closing the mouth corresponds to the gape cycle. Note the stereotypy of the magnitude of gape movements (parameter 1 and 2) in the LLM, the flexibility of the gape movements (parameter 1–4) in the MMM, the stereotypy of the hyobranchial movements (parameter 13,14, and 17) in the MMM, the stereotypy of the gape movements (parameter 1,2,3, and 5) in the PMM, and the flexibility of hyobranchial movements (parameter 13–15) in the PMM

Table 2 shows the statistical analysis of the kinematic parameters of food processing in the three morphotypes. Some significant changes concern the duplication of the vertical hyobranchial magnitude of the PMM compared to the MMM (compare with Fig. 4h and i), the duplication in gape magnitude from the MMM to PMM (compare Fig. 4b and c), and the significantly higher mean mandible acceleration from peak gape opening to reaching maximal gape-closing speed in the LLM compared to the MMM. The durations of the gape and vertical hyobranchial movement cycle are the same across all morphotypes.

**Table 2:** Statistical analysis of intraoral food processing kinematics in *I. alpestris*

Kinematic variable	Kinematic parameter	All Morphotypes		LLM vs. MMM		LLM vs. PMM		MMM vs. PMM	
		Kruskal-Wallis H	*p*-value	Mann-Whitney U	*p*-value	Mann-Whitney U	*p*-value	Mann-Whitney U	*p*-value
Gape	Opening	78.08	0.00*	−21.42	0.32	−80.09	0.00*	−58.67	0.00*
	Closure	79.09	0.00*	−17.58	0.55	−78.61	0.00*	−61.03	0.00*
	Opening duration	44.82	0.00*	−57.24	0.00*	−73.85	0.00*	−16.61	0.23
	Closure duration	30.18	0.00*	60.29	0.00*	59.85	0.00*	−0.43	1.00
	Closure acceleration	17.15	0.00*	51.24	0.00*	22.12	0.12	−29.12	0.00*
	Open-close duration	48.69	0.00*	−61.43	0.00*	−77.14	0.00*	−15.72	0.28
	Cycle duration	3.56	1.00	n/a	n/a	n/a	n/a	n/a	n/a
Vertical hyobranchial movement	Elevation	32.10	0.00*	−10.04	1.00	−49.48	0.00*	−39.45	0.00*
	Depression	64.27	0.00*	−18.06	0.52	−71.98	0.00*	−53.92	0.00*
	Elevation duration	18.37	0.00*	−49.55	0.00*	−45.79	0.00*	3.76	1.00
	Depression duration	33.78	0.00*	70.21	0.00*	23.89	0.09	−46.32	0.00*
	Cycle duration	5.22	0.88	n/a	n/a	n/a	n/a	n/a	n/a

Statistical analysis was calculated using Kruskal-Wallis 1-way ANOVA and only performed on parameters present in all morphotypes. *P*-values were Bonferroni adjusted to account for multiple testing; significant *p*-values are indicated by asterisks

### Ordination analysis of processing kinematics

**Fig. 5:**
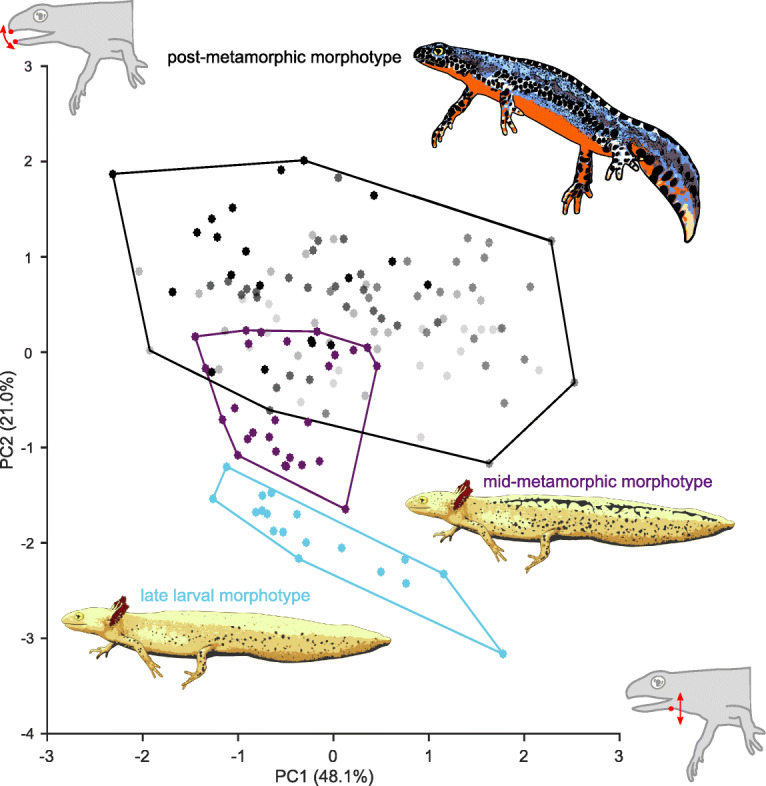
Scatterplots of the first two principal components. The principal components (PC1 and PC2) derived from 5 out of 12 kinematic parameters and illustrate differences between the three processing modes in kinematic space. Light blue, processing in the late-larval morphotype (LLM); purple, processing in the mid-metamorphic morphotype (MMM); and black, processing in post-metamorphic morphotypes (PMM) (5 shades of grey code the post-metamorphic individuals). The convex hulls display the largest possible area which contains all observations of the respective feeding mode. PC1 explains 48.1% of the total variance and is mostly defined by hyobranchial parameters, while PC2 explains 21.0% of the total variance and is most strongly defined by gape parameters (Table 3). Note that the MMM and PMM show overlap, while LLM and MMM as well as LLM and PMM show no overlap in kinematic space

A principal component analysis (PCA) was performed to analyze how the processing kinematics of the three morphotypes relate to each other and to visualize differences. Distribution of the chewing cycles among the processing modes and morphotypes on the first two principal components axes are shown in Fig. 5, and the loadings of the kinematic parameters on principal component 1 and 2 (i.e., PC1 and PC2) are given in Table 3. Hyobranchial kinematics load more strongly on PC1 while mandible kinematics loaded more strongly on PC2. Processing in PMM and LLM are separated in kinematic space with no overlap, but MMM processing overlaps with PMM while coming close to (i.e., being similar to) LLM processing.

**Table 3:** Loadings of processing parameters on the first two principal components (PC1 and PC2)

Parameter	PC1	PC2
(18) Duration hyobranchial movement cycle	0.836^a^	−0.123
(15) Magnitude hyobranchial depression	0.779^a^	0.353
(14) Magnitude hyobranchial elevation	0.663^a^	0.480
(1) Magnitude gape opening	−0.036	0.872^a^
(3) Duration gape opening	0.300	0.699^a^
Total variance explained (%)	48.1	21.0

Parameters marked with ^a^load strongly (> 0.5) on each respective principal component. Note that parameters connected to hyobranchial movement load more strongly in PC1 while parameters connected to gape movements load more strongly on PC2

The coefficient of variation (C_V_) was calculated for each kinematic parameter (Table 1) in order to quantify the stereotypy of the processing behavior of each morphotype [55]. The stationary gape phase (i.e., parameter 6) was only part of the processing mechanism in the LLM and parameters concerning vertical cranial flexion (8–12) could only be analyzed for the PMM. Consequently, these parameters were excluded for comparison.

### Stomach content analysis

**Fig. 6:**
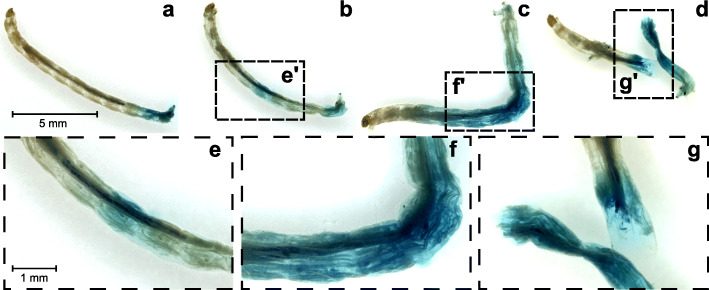
Lake fly larvae (*Chironomidae*) after intraoral processing. **a** Control specimen, and (**b**-**c**) processed larvae. The processed specimen exhibited (**b**) minor injuries, (**c**) major injuries, and (**d**) ruptures. Methylene blue staining highlight structural damages in the cuticle. Images **e**-**g** show details of the image sections **e**'-**g**'. Note that all samples (including the control) have a blue colored posterior area, probably due to the anal opening

Post-metamorphic newts used in stomach content analysis applied suction feeding to ingest lake fly larvae (*Chironomidae*). After ingestion, the newts used cyclic processing movements involving ventral cranial flexion and mouth opening accompanied by hyolingual elevation. Microscopic examinations of the processed lake fly larvae extracted from the stomachs of freshly euthanized newt specimens revealed clear lesions and other structural damage. Lesions were recognized by intensified methylene blue staining, which gradually attenuated along the unharmed part of the prey (Fig. 6b - d). By contrast, unprocessed lake fly larvae (control) only showed blue coloration in the posterior most region (Fig. 6a) and no structural damage. From a total of 100 processed lake fly larvae, 61 exhibited minor to major structural damage (Fig. 6b - c), 18 were ruptured (Fig. 1d) and 21 did not show evidence of damage (Fig. 6a).

## Discussion

We found distinct intraoral food processing kinematics (Fig. 5) and feeding apparatus morphologies (Figs. 1 and 2) in the three heterochronic morphotypes of the Alpine newt. Thus, this study shows that externally similar animals can have different internal anatomies, which in turn may result in different behaviors.

It was recently shown that metamorphosed salamandrid newts use loop-like movements of their hyolingual apparatus (i.e., tongue) to translate food across the palatal dentition (i.e., tongue-palate rasping) [21]. It has also been suggested that salamandrids with a larval morphology cannot employ the same processing mechanism as metamorphic animals because of morphological constraints, including the lack of a flexible tongue with sufficient internal movement-potential and diverging dentition patterns in larval morphotypes [15, 16]. To address the question of how processing differs between larval and metamorphosed salamandrid morphotypes of the same species, we examined heterochronic morphotypes of adult Alpine newts.

Our data support the hypothesis that larval salamandrids process their food differently than metamorphic salamandrids, as we observed many differences in prey processing behavior across heterochronic morphotypes (Table 1 and Fig. 5). For example, the late-larval morphotype (LLM) was the only morphotype to exhibit a stationary phase after the gape cycle and the post-metamorphic morphotype (PMM) was the only morphotype to show cranial flexion during processing (Fig. 4 and Table 1). Even if only kinematic parameters are compared that apply to all morphotypes, the mid-metamorphic morphotype (MMM) differed in 6 out of 12 kinematic parameters from the LLM, the PMM in 8 out of 12 kinematic parameters from the LLM and the PMM differed in 6 out of 12 kinematic parameters from MMM – suggesting that each morphotype could potentially use a different food processing mechanism.

The PMM of the Alpine newts used its tongue to cyclically and rhythmically drive the food against the vomerine dentition on the palate (Video 1 and Fig. 3c), very similar to movement patterns reported for the crested newt [21], that uses tongue-palate rasping to process prey. In fact, our stomach content analysis revealed that processing in *I. alpestris* caused substantial mechanical damage to the food objects (Fig. 6). Tongue-palate rasping in the PMM was characterized by relatively flexible tongue movements (C_V_ between 0.31–0.53, except for the relatively stereotypical hyobranchial cycle duration with 0.23), which may indicate that the tongue movements must be flexibly fine-tuned during processing. Similar to the PMM, the MMM showed evidence of a tongue-palate rasping mechanism being used as during processing the tongue was used to move the prey across the palate (Video 2) and haemolymph occasionally left the mouth (Video 3). The tongue-palate rasping mechanism of the MMM deviated from that of the PMM in that it showed a modified tongue motion pattern (compare Fig. 4b and h to c and i) and small and sporadic cranial movements (Fig. 4e). Tongue-palate rasping in the MMM was characterized by relatively stereotypical movements of the tongue (C_V_ between 0.28–0.35, excluding the relative flexible duration of hyobranchial elevation with 0.45). With regard to the switch from chewing (LLM) to tongue-palate rasping (MMM), this could suggest that a relatively stereotypical motion sequence is used first when mastering a new behavior pattern, while this motion sequence can become more flexible during ontogenesis (as seen in the PMM).


**Additional file 1: Video 1.** High-speed movie from a lateral perspective, showing a post-metamorphic *I. alpestris* processing a lake fly larva (Chironomidae) under water. Three consecutive processing cycles can be observed. Note how the tongue cyclically rasps the prey along the palate. The movie was recorded at 500 Hz and is played back at 50 Hz, which corresponds to 10% of the actual speed.


**Additional file 2 Video 2.** High-speed movie from an anterio-lateral perspective, showing a mid-metamorphic *I. alpestris* processing a lake fly larva (Chironomidae) under water. Five full processing cycles can be observed, cycle 1, 4, and 5 are mixed, or immediately followed, by transport movements. Note how the tongue cyclically rasps the prey along the palate. The movie was recorded at 500 Hz and is played back at 50 Hz, which corresponds to 10% of the actual speed.


**Additional file 3 Video 3.** High-speed movie from a lateral perspective, showing a mid-metamorphic *I. alpestris* processing a lake fly larva (Chironomidae) under water. Five full processing cycles can be observed, cycle 3 is immediately followed by a transport movement. Note how in every except the third cycle the haemolymph of the prey leaves the mouth during processing (i.e., an indication of structural damage). The movie was recorded at 500 Hz and is played back at 100 Hz, which corresponds to 20% of the actual speed.

The LLM used a processing mechanism with a limited mouth opening, which initially prevented us from determining how food was processed. However, further observations showed that the LLM chewed its food because the prey occasionally protruded from the mouth and therefore made clear how the jaws act upon the prey (Video 4). We could distinguish the post-ingestion behavior (i.e., jaw and hyobranchial movements) into prey transport (characterized by hyobranchial depression during gape opening [17–19]) and rhythmic food processing (characterized by hyobranchial elevation throughout or during some of the gape opening cycle [21, 23]). Food-processing kinematics in the LLM involved the highest mean gape-closure acceleration (Fig. 4a – c and Table 1). As the mandibles of all morphotypes are of approximately the same size and therefore likely have approximately the same mass, the finding that the LLM showed the highest mean gape-closure acceleration might suggest that they exhibit the highest bite force. This, in turn, supports the idea that the dentition of the mandible is directly involved in intraoral food processing (i.e., chewing). We use the term bite force to describe the result of the action of the mandible elevator muscles modified by the craniomandibular biomechanics [56] and thus the force that the mandible can transmit onto an object in the oral cavity (therefore not merely equivalent to adductor muscle force). Additionally, one of the most striking characteristics of the LMM cranio-mandibular anatomy is its overbite, causing dentition on the mandible to occlude between the two functional upper jaw systems, creating an effective shearing bite against the palate (Fig. 1b and c). Consequently, the morphology of the LLM supports the idea that it chews its food using the tooth-bearing mandible (Fig. 1) to pierce the prey against the palate (i.e., ‘mandible-palate clenching’) while the tongue and dentition on both functional upper jaws hold the prey in place. The kinematic profiles support this assumption as initial gape opening is followed by hyobranchial elevation, which potentially act to position and hold the prey in the area of the occlusal surface on the palate, before the mandible accelerates towards the palate (Fig. 4a and g) to bite the prey.


**Additional file 4 Video 4.** High-speed movie from a lateral perspective, showing a late-larval *I. alpestris* processing a lake fly larva (Chironomidae) under water. Five full processing cycles can be observed, cycle 5 is mixed with a transport movement. Note how the lower jaw is used to bite the prey. The movie was recorded at 500 Hz and is played back at 100 Hz, which corresponds to 20% of the actual speed.

Externally, the processing behavior of LLM showed striking similarities with the chewing behavior of another paedomorphic salamander, *Siren intermedia*. It was shown using high-speed x-ray analyses that *S. intermedia* use its mandible to rasp the prey across the dentition of the palate [23]. Larval Alpine newts, however, chew their food using ‘simple’ arcuate movements of the mandible (i.e., opening-closing), and switch from chewing to tongue-palate rasping during ontogeny. Tongue-palate rasping appears to become the main food processing mechanism before the tongue is completely remodeled during metamorphosis (Fig. 2b, Fig 3b, and Video 2). The behavioral shift from mandible-palate interactions (i.e., chewing) to tongue-palate rasping corresponds with the key morphological changes between morphotypes. Whereas in the LLM the teeth of the V-shaped mandible impinge on the palate between the dentition of both functional upper jaw systems (allowing shearing action), the U-shaped mandible of the MMM and the PMM would occlude with more latero-caudal elements of the "primary" upper jaw (i.e., maxilla) (Fig. 1 f-g and j-k) upon jaw closing. The change in mandible shape might prevent mandible-based processing in a progressed mid-metamorphic morphotype as (i) there is only a limited occlusal surface between the mandible and the latero-caudal part of the "primary" upper jaw for chewing and (ii) food loading might be insufficient, given that there is no bone bridge between the rear end of the primary maxilla and the anterior quadrato-squamosal region [57]. Food processing is often argued as being important for the immobilization and break-down of food items before swallowing [58–61] so salamanders might need alternative food processing mechanisms once their mandible outgrows its chewing function. Interestingly, this flexible switch from one processing mechanism to another took place in a single stage of development as both morphotypes (LLM and MMM) were paedomorphic. This appears to reflect the complex morphological life cycle of many salamanders, in which there may be different morphological expressions of paedomorphism, with the morphology of some paedomorphic animals being very similar to that of metamorphs [30]. Not least for this reason, we suspect that the sequence of behaviors we observed could be typical for the development of many salamanders.

It has been previously hypothesized that salamanders show a phylogenetic trend of evolving tongues with greater protrusion potential, increased freedom of the branchial arch in relation to the hyoid arch, and that tongue prehension might have evolved from a intraoral "manipulation" function [28]. Intraoral "manipulation", which was originally understood as pure transport behavior [19, 28], has recently been interpreted as a continuum of processing and transport behavior in terrestrial salamanders [21]. In line with Regal’s idea, we found a concurrent ontogenetic process of remodeling in the tongue apparatus. As during the newt ontogeny the tongue develops from a bulky relatively inert system (i.e., hyobranchial system) with small protrusion ability in the LLM (Fig. 1d and Fig. 2a) to a delicate and relatively mobile system (i.e., hyolingual system) with greater protrusion ability in the PMM (Fig. 1l and Fig. 2c). The LLM hyobranchial system has a muscular anatomy that creates motion-potential in all directions of the median plane. However, tongue protraction is limited to geniohyoid and ceratomandibularis muscles which act as the primary tongue protractor complex in larval salamanders (Fig. 2a). During the metamorphosis in the MMM, the branchiohyoideus externus and subarcualis rectus 1 muscles are rearranged to functionally suspend the branchial arch on the paired ceratohyal (i.e., hyoid arch) (Fig. 2b). This muscle rearrangement enables a more effective protraction of the branchial arch, since it can now be moved by the suspension on the hyoid arch and thus pulled further anteriorly (Fig. 2b). This secondary tongue protractor complex allows the tip of the tongue to be ejected out of the mouth which has been described for post-metamorphic salamandrids [33] and in turn is the functional basis for tongue-palate rasping [21]. Our data suggest that aquatic salamandrid larvae begin to use their tongue for intraoral food processing (Fig. 3b and Video 2) as soon as the mandibular reorganization prevents them from chewing but their tongue morphology enables improved protraction during development. Thus, we hypothesize that salamanders that are able to protract their tongue effectively and have a metamorphic palatal dentition (Fig. 1c and g) are potentially able to combine these elements to achieve tongue-palate rasping. Consequently, it is likely that tongue-palate rasping is the general processing mechanisms in salamanders with a metamorphic feeding apparatus morphology. Additionally, our data support Regal’s hypothesis that tongue prehension likely evolved from a "manipulation" function of the tongue [28] as our animals mastered tongue-palate rasping before they were apt to leave the water and thus before they used their protractible tongue to catch prey.

Mid-metamorphic Alpine newts develop the ability to rasp a food item against the palatal dentition and engage in tongue-palate rasping due to rearrangements of the branchiohyoideus externus and subarcualis rectus 1 muscles during metamorphosis. At the same time mid-metamorphic morphotypes also retain the ability to forcefully elevate their tongue using the levator hyoideus muscle (Fig. 2b). The tongue of the post-metamorphic morphotype loses this muscular connection to the skull (i.e., levator hyoideus) and its motion is limited to elevation based on muscles spanning the mouth floor and the hyobranchial system laterally [35]. As a result, the tongue is likely to lose the ability to forcefully press a food against the palatal dentition, possibly reducing the effectiveness of tongue-palate rasping. It had been hypothesized that the coordination between hyolingual motion and depression of the skull may aid food processing efficacy in post-metamorphic salamandrids [21]. Coordinated head and hyolingual movement patterns also appear in post-metamorphic Alpine newts (Fig. 4f, i), but not in the mid-metamorphic stage, suggesting that coordinated depression of the skull and hyolingual movements might be a compensatory behavior for the loss of the levator hyoideus (Fig. 2c).

From an evolutionary perspective the findings presented here might shed light on the fish-tetrapod transition (water-land transition) of early tetrapods. While tongue and jaw kinematics are similar across amniotes [24, 25, 62], food processing in salamanders shares traits with both fish and amniotes [21]. Accordingly, salamanders may be a good analog model to reveal functional changes in feeding behavior across the fish-tetrapod transition [41]. From this point of view, the morphological and behavioral differences between the two aquatic larval stages (LLM and MMM) could reflect analogous changes in the early tetrapods. In particular, the present study shows that the MMM – a stage with both larval and post-metamorphic traits and without a freely movable tongue - utilizes a new feeding mechanism (tongue-palate rasping) before the presumed morphological adjustments for this function have fully developed and while the animal remains fully aquatic. It is possible that behavioral changes may have preceded obvious morphological evolution of the feeding system across the fish-tetrapod transition, resulting in new feeding mechanisms. Thus, understanding the timing of changes in feeding mechanism across the transition may require precise quantification and characterization of morphology as well as rigorous biomechanical testing, which can reveal biomechanical differences in similarly-shaped structures [63]. Furthermore, our results support findings from previous studies that morphological and behavioral changes facilitated the evolution of “terrestrial style feeding” in early tetrapod taxa that were still primarily aquatic [64–67].

## Conclusions

We found differences in the skeleton, soft tissue and food processing kinematics between the late-larval morphotype (LLM) and the mid-metamorphic morphotype (MMM), suggesting previously unappreciated diversity between superficially similar paedomorphic stages. Further, our data show that prey processing kinematics differ between all three morphotypes (late-larval, mid-metamorphic and post-metamorphic morphotype) in the Alpine newt, contrary to the previously established pattern of stereotypy of prey capture or intraoral transport kinematics for this species. Our data indicate a degree of plasticity not previously demonstrated in the ontogeny of intraoral food processing behaviors. Based on a similar development in the feeding apparatus morphologies of most larval salamanders, our data also suggest that salamanders may undergo similar food processing ontogenies in general. Additionally, we found, that salamanders that are able to protract their tongue effectively and have proper palatal dentition, are potentially equipped to use tongue-palate rasping. Consequently, it is likely that tongue-palate rasping is a generalized pattern in salamanders with a metamorphic feeding apparatus morphology (i.e., MMM and PMM). Finally, the present study might allow some parallels to be drawn about the evolution of terrestrial feeding in early tetrapods. In analogy to salamanders, early tetrapods might have evolved new feeding mechanisms in their aquatic environments and these functional innovations later might have paved the way for terrestrial feeding mechanisms.

## Methods

### Specimens and animal care

The paedomorphic and metamorphic specimens used in this study were collected in September of 2012 from an artificial irrigation reservoir in the Province of Bolzano (South Tyrol, Italy) under collection permit No. 63.01.05/120963, granted by the local government of the Province of Bolzano. For further information on the pond and the paedomorphic character of the specimens, see [68]. The natural prey spectrum of metamorphic and paedomorphic *Ichthyosaura alpestris* is very broad. In the aquatic habitat, the Alpine newt feeds on insect larvae (e.g. *Chironomidae*), small crustaceans and amphibian eggs or larvae [69, 70].

Kinematic analyses were conducted using five post-metamorphic individuals (five PMM) and two paedomorphic individuals (one LLM, one MMM). The kinematic analyses of the LLM and the MMM were therefore limited to the repetitions of one specimen each (for details to the number of repetitions see “High-speed recording and kinematic analysis”). The SVL of paedomorphic specimens (43 and 45 mm) did not differ significantly from the SVL of post-metamorphic specimens (44.6 ± 3.4 mm). The animals were group-housed with both paedomorphic newts in one aquarium (60 × 30 × 40 cm; (length × width × depth)) and the post-metamorphic newts in a larger aquarium (120 × 60 × 40 cm). The animals were kept at 20 ± 2 °C temperature, 12/12 h photoperiod and were exclusively fed with lake fly larvae (*Chironomidae*) a week before the recordings.

### High-speed recording and kinematic analysis

The newts were placed in a glass aquarium (30 × 12 × 20 cm) with a water level of approximately 10 cm. Paedomorphic and post-metamorphic individuals were fed with lake fly larvae (*Chironomidae*). A chessboard pattern was placed in the background of the aquarium to allow calibration of the videos. The test setup was illuminated with reduced heat emission spotlights (VD-7000 LP; Vision Devices GmbH, Metzingen, Germany). A high-speed camera (Photron FASTCAM model 100KC; Photron, Tokyo, Japan) was used to record the feeding events at 500 fps with a 1024 × 512 pixel frame format. Recordings of paedomorphic feeding trials was conducted using a 60 mm macro lens while a 50 mm standard lens was used for post-metamorphic trials. A total of 49 recordings from paedomorphic and 50 recordings from post-metamorphic newt feeding were acquired.

**Fig. 7:**
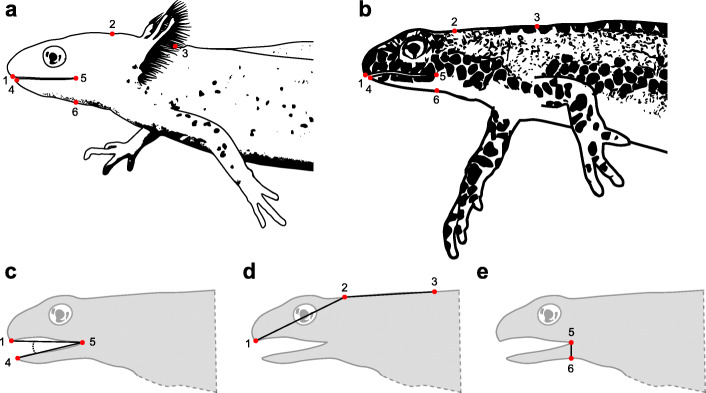
Landmark overview for kinematic analyses. **a** Paedomorphic and (**b**) post-metamorphic *I. alpestris* with landmarks used for kinematic analyses. Lower row depicts the calculation of (**c**) gape, (**d**) vertical cranial flexion and (**e**) vertical hyobranchial displacement. Abbreviations: (1) tip of the upper jaw, (2) back of the head, (3) reference point on the back approximately over the shoulder girdle (4) tip of the mandible, (5) corner of the mouth, and (6) point ventral to the corner of the mouth which lowers as the hyobranchium is depressed

Recordings for kinematic analyses were selected according to overall sharpness (focus on the specimen) as well as specimen orientation. Landmark tracking was carried out using Simi Motion 8.0.0.315 software (Simi Reality Motion Systems GmbH, Unterschleißheim, Germany). Three component motions - gape cycle, vertical cranial flexion, and dorso-ventral hyobranchial movement - were analyzed. To do so, we tracked six landmarks: (1) tip of the upper jaw, (2) back of the head, (3) reference point on the back approximately over the shoulder girdle (4) tip of the mandible, (5) corner of the mouth, and (6) point ventral to the corner of the mouth which lowers as the hyobranchial apparatus is depressed (Fig. 7a-b). Every fifth frame was used for manual landmark tracking, the missing intermediate time steps were spline interpolated, and areas of the resulting motion graphs that showed very small movements were locally smoothed using a moving average filter of the tracking software. Using the smoothing and interpolation functions of the tracking software allowed confirmation of the markers’ positions on their specific landmarks. In total, 105 processing cycles of post-metamorphic newts (PMM) and 45 processing cycles of paedomorphic newts (27 MMM and 18 LLM) were analyzed. We used trigonometry on the 2-D landmark coordinates to calculate the kinematic parameters gape (Fig. 7c), vertical cranial flexion (Fig. 7d), and vertical hyobranchial movement (Fig. 7e) in Excel (Microsoft Corporation, WA, USA). Subdivision of the kinematic profiles into component motion cycles was achieved using a custom graph analyzer tool for MATLAB R2019b (The Mathworks, Inc., Natick, MA, USA). A cycle was defined as a movement event that contained three extremes: two low or high points and the opposite point. The custom-written script additionally computed the 18 kinematic parameters out of the kinematic variables by using the high and low point information.

### Statistical analysis and ordination approach

The aim of the statistical analysis was to test the kinematic parameters for differences between the morphotypes (LLM, MMM and PMM). Since the parameters violated the assumptions for parametric tests, nonparametric statistics were carried out. To determine if the kinematic parameters differed between morphotypes, we performed a Kruskal-Wallis 1-way ANOVA. The sequential pairwise multiple comparisons (i.e., Mann-Whitney U tests), using ranks based on considering all samples, not just the two samples that are currently involved in a comparison [71], were performed to determine where the differences are among the morphotypes. All significance values were Bonferroni adjusted to account for multiple testing.

The aim of the ordination approach was to visualize how the processing kinematics of the three morphotypes are related and thus by implication to highlight their differences. First, processing parameters that do not apply to all morphotypes were excluded (7, 9–13 of Table 1). Subsequently, seven dimension reductions were performed prior to the principal component analysis (PCA) to remove processing parameters (2,4–8, 16–17 of Table 1) which do not load strongly (< 0.5) on any of the components. The PCA was performed on the correlation matrix and the resulting Anderson-Rubin factor scores were saved in order to show the effects of (i) individual, (ii) heterochronic state, and (iii) processing mode on the total variance. The factor scores of the PCA were plotted with the related convex hulls, displaying the biggest possible area, which contains all observations of the respective feeding mode. The principal component analysis was performed using SPSS 25 (IBM Corporation, Armonk, NY, USA).

### Stomach content analysis

Once the feeding trials and the kinematic analysis were performed, we fed the post-metamorphic animals with lake fly larvae ad libitum. They were presented with a multitude of lake fly larvae, which they ingested and processed one to several at a time. After feeding, the animals were anaesthetized and subsequently euthanized by immersion in an aqueous solution of 0.5% MS222, buffered to pH 7.0. The stomachs were removed and voided post-mortem and the stomach contents were fixed in 70% ethanol for 1 week. The processed lake fly larvae were stained using methylene blue (1 min) and then washed with 70% ethanol. The processing injuries were recorded using a digital microscope (Keyence, VHX-2000; Keyence Corporation, Osaka, Japan). The paedomorphic samples had to be euthanized after a fungal infection before data collection was completed; thus no paedomorphic stomach contents could be analyzed.

### Anatomical analysis (μCT)

The musculoskeletal components of the feeding apparatus of paedomorphic and post-metamorphic specimens were reconstructed from μCT scans [15]. Euthanized specimens were fixed in 4% formaldehyde for 1 month, dehydrated in a graded series of ethanol, immersed for 2 weeks in an alcoholic iodine solution, rinsed in absolute ethanol and securely mounted in Falcon tubes to avoid motion artifacts in the scans. Scans of the entire paedomorphic specimens were acquired using a μCT scanner (SkyScan 2211; Bruker, Billerica, MA, USA) with a source voltage of 100 kV, an electric current of 180 μA, a 0.5 mm Titan filter, and an isometric voxel resolution of 8.00 μm. X-ray projections were then reconstructed in NRecon Reconstruction Software 1.7.3.1 (Micro Photonics, Allentown, PA) with an automatic beam hardening correction factor of 45%. For the post-metamorphic specimen only scans of the head region were acquired using a μCT scanner (SkyScan 1174) with a source voltage of 50 kV, an electric current of114 μA, a 0.5 mm aluminum filter, and an isometric voxel resolution of 7.39 μm. X-ray projections were also reconstructed in NRecon Reconstruction Software. Volume rendering of the μCT scans was performed using the Amira 6.4 software package (https://www.fei.com/software/amira). Based on tomographic image data, we threshold-segmented bones and used manual segmentation for muscles, cartilage and teeth. Both paedomorphic specimens are kept in the State Museum of Natural History Stuttgart (SMNS 16344 and SMNS 16345).

## Data Availability

The datasets during and/or analyzed during the current study available from the corresponding author on reasonable request.
